# Biguanides in combination with olaparib limits tumorigenesis of drug‐resistant ovarian cancer cells through inhibition of Snail

**DOI:** 10.1002/cam4.2738

**Published:** 2019-12-21

**Authors:** Qiong Wang, Vanessa M. López‐Ozuna, Tahira Baloch, Joanne Bithras, Oreekha Amin, Roy Kessous, Liron Kogan, Ido Laskov, Amber Yasmeen

**Affiliations:** ^1^ Segal Cancer Center Lady Davis Institute of Medical Research McGill University Montreal Quebec Canada; ^2^ Department of Gynecology and Obstetrics Guangzhou Women and Children's Medical Center Guangzhou China; ^3^ Division of Uterine Vascular Biology Guangzhou Institute of Pediatrics Guangzhou Women and Children’s Medical Center Guangzhou China; ^4^ Division of Gynecologic Oncology Jewish General Hospital McGill University Montreal Quebec Canada; ^5^ Department of Experimental Surgery McGill University Montreal Quebec Canada

**Keywords:** combined therapy, EMT, metastatic ovarian cancer, PARP inhibitors, transcription factor

## Abstract

Ovarian cancer is the most lethal gynecological malignancy. Currently, new chemotherapeutic strategies are required to improve patient outcome and survival. Biguanides, classic anti‐diabetic drugs, have gained importance for theiri antitumor potency demonstrated by various studies. Olaparib is a PARP inhibitor approved for maintenance therapy following platinum‐based chemotherapy. Furthermore, Snai1, a transcription factor that works as a master regulator of the epithelial/mesenchymal transition process (EMT) is involved in ovarian cancer resistance and progression. Here we aimed to demonstrate the possible cross talk between biguanides and Snail in response to olaparib combination therapy. In this study, we have shown that while in A2780CR cells biguanides reduced cell survival (single treatments ~20%; combined treatment ~44%) and cell migration (single treatments ~45%; biguanide‐olaparib ~80%) significantly, A2780PAR exhibited superior efficacy with single (~60%) and combined treatments (~80%). Moreover, our results indicate that knock‐down of Snail further enhances the attenuation of migration, inhibits EMT related‐proteins (~90%) and induces a synergistic effect in biguanide‐olaparib treatment. Altogether, this work suggests a novel treatment strategy against drug‐resistant or recurrent ovarian cancer.

## Graphical abstract

Biguanides in combination with PARP inhibitors synergistically reduce EMT, proliferation and survival of ovarian drug‐resistant cancer cells.

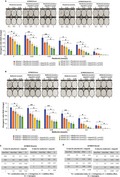

## 1 INTRODUCTION

Ovarian cancer (OC) is the most lethal gynecologic cancer. More than 75% of affected women are diagnosed at an advanced stage and less than one‐half of the patients survive for more than 5 years after diagnosis. High‐grade serous ovarian carcinoma (HGSOC) is the most commonly diagnosed. Currently, the standard care of treatment consists of debulking surgery followed by chemotherapy, however, approximately 70% of them will eventually undergo a relapse, metastasis and become drug‐resistant.^1^ This information highlight the need of identifying new molecular targets and the implementation of new combination of treatments.^2^

Metastatic spread is one of the major problems in OC. This process involved ovarian cancer cells to undergo an epithelial‐to‐mesenchymal transition process (EMT) by which cells lose their cellular adhesion capacity and gain migratory properties. The initial steps of EMT are regulated by several transcription markers. Snail has been suggested as an important effector through negative regulation of E‐cadherin by destabilization of adherent junctions (Bolos et al, 2003, Peinado et al, 2004). Furthermore, Snail has been described as major determinant for ovarian cancer growth, invasiveness and metastasis (Jin et al, 2010; Kurrey et al, 2005, Taki et al, 2018, Abdulkhalek et al, 2014).

Poly ADP ribose polymerase inhibitors (PARPi) are presently approved as maintenance therapy following platinum and taxol chemotherapy. PARPi function by blocking PARP1 protein, inducing synthetic lethality in HGSOC containing mutations in genes involved in the homologous recombination pathway of DNA repair (HR).^3^, ^4^, ^5^, ^6^, ^7^, ^8^ Olaparib was the first PARPi to be introduced as a maintenance treatment for ovarian cancer patients.^9^

Biguanides are recommended as a first‐line therapy for patients with type II diabetes, however they have gained significant attention as a potential anticancer agent.^10^, ^11^, ^12^ Metformin was proved to induce ovarian cancer cell cytotoxicity in combination with cisplatin.^13^ Moreover, in vitro and in vivo studies have proved to induce apoptosis, inhibit angiogenesis and metastatic spread of ovarian cancer.^14^, ^15^, ^16^, ^17^ In addition, phenformin, another biguanide, is 50 times as potent as metformin, which has been recently theorized to have antitumorigenic efficacy. Furthermore, a recent study has shown phenformin to inhibit ovarian cancer tumor growth in vitro and in vivo and induct cell cycle arrest and apoptosis (Jackson et al, 2017).

In this study, we explored the effects of biguanides alone or in combination with olaparib in drug‐resistant ovarian cancer cells. In addition, we evaluated the potential effects on migration and survival by targeting the transcription factor Snail.

## 2 MATERIALS AND METHODS

### 2.1 Cell lines and reagents

A2780 parental (PAR) and A2780 cisplatin resistance daughter clone (CR) cells were provided by Dr Seftor (Northwestern University, Chicago). All the cells lines were authenticated by short tandem repeat (STR) profiling using DNA sequencing. This analysis was performed at the University of Colorado which has extensive experience in evaluation of gynecological cell lines.^18^ A minimum of 95% of similarity between a reference profile and our cell lines was observed. Cell lines were cultured in Roswell Park Memorial Institute (RPMI) 1640 medium, supplemented with 5% fetal bovine serum (FBS), 10mM Hepes and antibiotics (penicillin 100 U/mL, streptomycin 0.1 mg/mL and amphotericin B 0.25 μg/mL) and maintained at 37°C in a humidified incubator with 5% CO_2_. Phenformin Hydrochloride and Metformin Hydrochloride were bought from Toronto Research Chemicals. Olaparib (AZD2281) was obtained from AdooQ Bioscience.

### 2.2 Generation of stable cell lines

A2780CR cells were seeded in 6‐well flat‐bottom cell culture plates at a density of 0.25 × 10^6^ cells/well. Lipofectamine (Invitrogen) (1:1) was mixed with control shRNA and Snail shRNA separately in RPMI‐1640 with no FBS. Following 30 minutes of incubation at room temperature, both negative and Snail10‐2, shRNA (NM005985, sequence: CCGGTGCTCCACAAGCACCAAGAT.

GTCTCGAGACTCTTGGTGCTTGTGGAGCATTTTTTG) were added to their respective wells (Sigma Aldrich TRCN0000453110). The cells were incubated at 37°C for 5 hours. Pools of stably transfected cells were selected using 2mg/ml puromycin for up to a week, as previously described.^19^

### 2.3 Cell survival assays

A2780PAR, A2780CR/ CR shSnail 10‐2 and shVector cells were plated in 6 wells in duplicates. Cells were washed and fresh medium was added in the presence or absence of increasing doses of Metformin (0‐5 mmol/L)/ Phenformin (0‐1 mmol/L) alone or in combination with olaparib (0.1‐0.5 µM). Media containing the drug were refreshed on day 3. Dimethyl sulfoxide (DMSO) was used as a control. The experiment was discontinued when the clones reached 50 cells/clone in the DMSO‐vector wells (7 to 12 days). The colonies were fixed and stained with 1.5 mL of 6.0% glutaraldehyde and 0.5% crystal violet, and colonies were counted using a GelCount^TM^ (Oxford Optronix). The surviving fraction (SF) of cells was calculated as follows: SF = (number of colonies formed after treatment)/(number of cells seeded x plating efficiency), where the plating efficiency = (number of colonies formed in control)/(number of cells seeded).^20^ Drug interaction was assessed using the multiple drug effects analysis method of Chou and Talalay.^21^ This method quantitatively describes the interaction between two or more drugs, with combination index (CI) values less than 1 indicating synergistic interactions, values greater than 1 indicate antagonistic interactions, and values equal to 1 indicate additive interactions. Calculations of the CI values were performed with CompuSyn Software (ComboSyn, Inc).

### 2.4 Alamarblue assays

Cell proliferation assay was performed by alamarBlue staining. A2780PAR or A2780CR cells were plated at 1 × 10^4^ cells/well in 96‐well plates, cultured overnight, treated for 72 hours and analyzed using spectrophotometry at wavelengths of 562 and 600 nm according to the manufacturer's instructions (Bio‐Rad). Treatments included: phenformin (0‐1 mmol/L), metformin (0‐10 mmol/L) with/without Olaprib (0‐2 µmol/L).

### 2.5 Cell migration assay

Cell migration was examined using wound healing assay. A2780PAR, A2780CR/ CR shSnail 10‐2, and shVector cells were plated at 5 × 10^5^ cells/well in 6 well plates and grown to confluence in 24 hours. Then a scratch was performed on the confluent monolayer with a 200 µL pipette tip, followed by 3 washes with PBS and treatment for 24 hours. The wounded areas were photographed at 0 and 24 hours after scraping. The treatments included phenformin (0‐1 mmol/L), metformin (0‐5 mmol/L), olaparib (0‐2 µmol/L), phenformin (0‐1 mmol/L) + olaparib (0.1 µmol/L), phenformin (0‐1 mmol/L) + olaparib (0.5 µmol/L), metformin (0‐5 mmol/L) + olaparib (0.1 µmol/L) and metformin (0‐5 mmol/L) + olaparib (0.5 µmol/L).

### 2.6 Western blot assay

Lysates from A2780PAR, A2780CR/ CR shSnail 10‐2 and shVector cells, were collected and subjected to protein determination using Bio‐Rad protein assay kits (Bio‐Rad) and then to sodium dodecyl sulfate‐polyacrylamide gel electrophoresis (SDS‐PAGE) for protein separation. The proteins were transferred from the gels into nitrocellulose membranes (Bio‐Rad). After blocking with 5% milk at room temperature for 1 hour, the membrane was incubated at 4°C overnight with specific antibodies, including Snail (1:500 dilution), Vimentin (1:500 dilution), E‐Cadherin (1:500 dilution), N‐Cadherin (1:500), Twist (1:500 dilution), Slug (1:500 dilution), Fibronectin (1:500 dilution) and β‐Actin (1:5000 dilution). All antibodies were purchased from Cell Signaling Technology. Protein bands were detected using Clarity^TM^ Western ECL Substrate kit (Bio‐Rad) and visualized by exposure to X‐ray film. Bands were quantified using image J software, films were scanned and images were converted to 8 bit format. The area value of each was obtained, normalized to β actin, quantified and statistical analysis was performed.

### 2.7 Statistical analysis

Results are represented as means ± standard deviations of three independent experiments. To analyze the differences, one‐way ANOVA followed by Tukey's post ‐hoc test was applied and *P* < .05 was considered statistically significant.

## 3 RESULTS

### 3.1 Biguanides alone or in combination of low doses of olaparib inhibit cell survival

**Figure 1:**
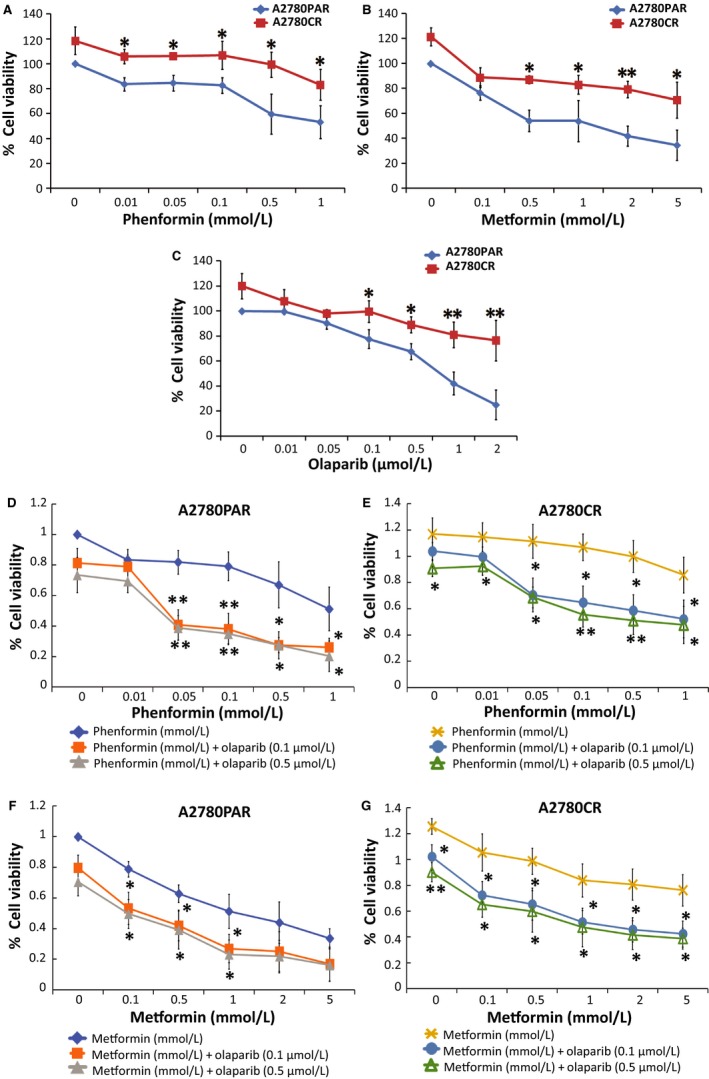
Biguanides alone and/or in combination with olaparib inhibit OC cell proliferation. A2780PAR and A2780CR ovarian cancer cells were treated for 72 hours and cell proliferation was determined using alamarBlue assays. (A) Phenformin (0‐1 mmol/L), (B) metformin (0‐5 mmol/L), (C) olaparib (0‐2 µmol/L), phenformin‐olaparib (D and E) and metformin‐olaparib (F and G). Data represent the mean ± SEM of triplicate of three independent experiments, statistical significance is represented by *(*P* < .05) and **(*P* < .01)

The effect of phenformin, metformin and olaparib as single treatments in OC cell viability was assessed using the alamarBlue assay. Two ovarian cancer cell lines, A2780PAR and their resistant clone A2780CR, were treated with different single treatment concentrations for 72 hours. As shown in Figure 1A‐C, phenformin, metformin and olaparib inhibit cell viability in a dose‐dependent manner after 72 hours (~55% in A2780PAR and ~20% in A2780CR). Next, we evaluated the effects of both biguanides in combination with olaparib (0.1 and 0.5 µmol/L). We found that the addition of olaparib enhances the effects of biguanides in the drug‐resistant clones and decrease even more its survival (~43% metformin‐olaparib *P* < .0082, ~45% phenformin‐olaparib *P* < .0009) (Figure 1D‐G). These results suggest that both biguanides and olaparib as single treatments are effective in inhibiting cell survival in A2780PAR as compared to the resistant cell line. Moreover, the combination of biguanides and olaparib potentiates the inhibition of cell survival in parental and resistant cells. We have observed similar results that are in agreement with the findings of the Hijaz et al.^22^

### 3.2 Biguanides show a synergistic effect with olaparib to reduce cell growth and survival

**Figure 2:**
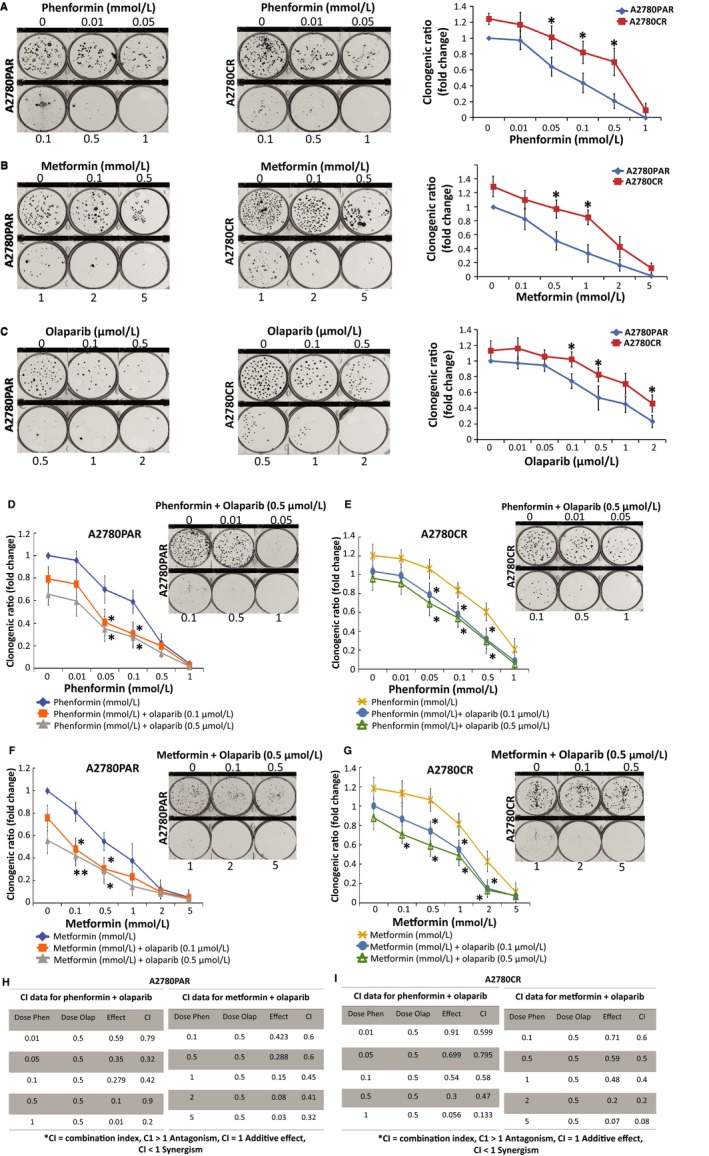
Biguanides alone or in combination with olaparib inhibit OC cell colony formation. A2780PAR and A2780CR ovarian cancer cells were treated for 7 days and survival was determined using clonogenic assays. (A) Phenformin, (B) metformin, (C) olaparib, (D and E) phenformin‐olaparib (F and G) and metformin‐olaparib. The evaluation of combination index for A2780PAR (H) or A2780CR (I) treated with phenformin or metformin and olaparib was calculated where CI < 1 indicates synergy between the two drugs and CI > 1 indicates an additive effect. Results are presented as mean ± SEM for triplicate of three independent experiments

Next, to corroborate the antitumorigenic capacity of both biguanides*,* a colony formation assay was employed. A2780PAR and its resistant clone A2780CR were treated with varying concentrations of phenformin (0‐1 mmol/L) (Figure 2A), metformin (0‐5 mmol/L) (Figure 2B) and olaparib (0‐2 µmol/L) (Figure 2C) as single treatment. Our results revealed that the treatments with phenformin, metformin and olaparib could inhibit the colony formation capacity of A2780PAR cells and to a lesser extent A2780CR cells in a dose‐dependent manner.

Next, we observed that the addition of olaparib to the biguanides (Figure 2D‐G) as combined therapy, potentiates the inhibition of cell growth in A2780PAR and its resistant clone A2780CR as compared with single treatments (Figure 2A‐C). Specifically, both cell lines showed a lower clonogenic ratio after cotreatment with olaparib (0.1 and 0.5 µmol/L). To further determine the nature of the interaction we used the multiple drug effects analysis method by Chou and Talalay showing high synergistic effect (CI < 1) (Figure 2H‐I).

### 3.3 Combination of biguanides and olaparib significantly inhibits cell migration and invasiveness of OC cells

**Figure 3:**
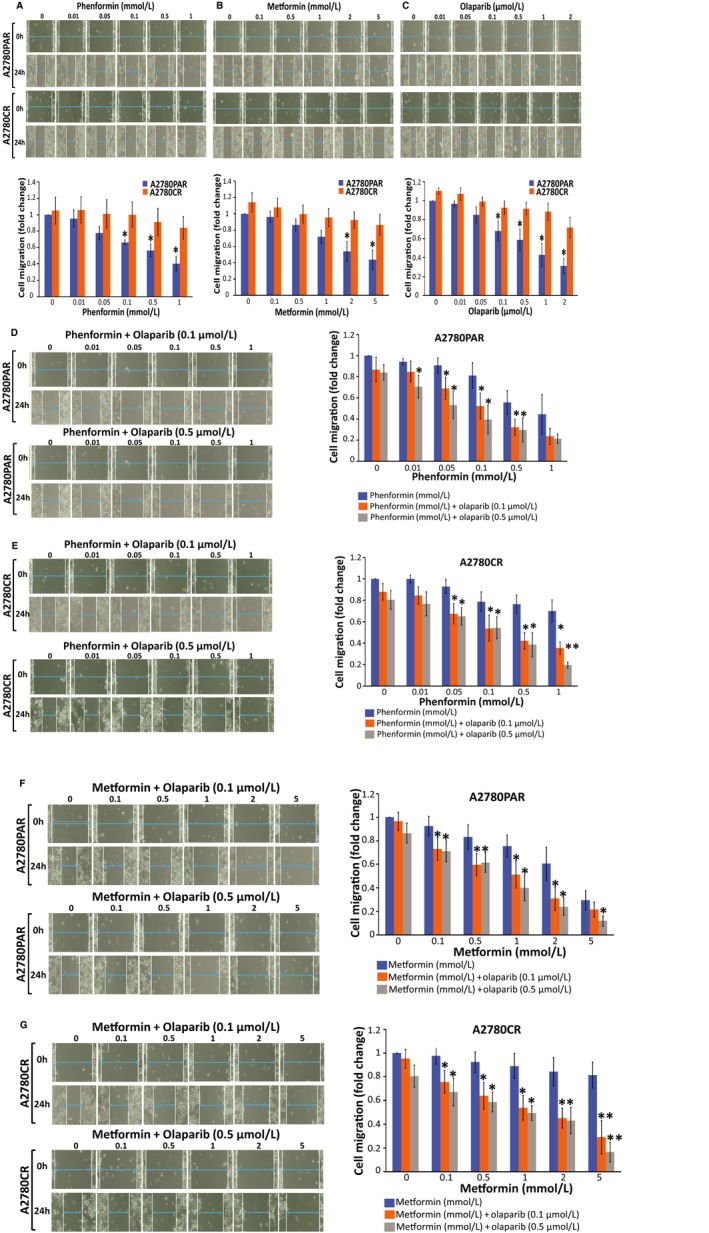
Biguanides alone or in combination with olaparib inhibit OC cell migration. A2780PAR and/or A2780CR ovarian cancer cells were treated for 24 hours and wound healing assays were performed to evaluate cell migration. (A) Phenformin, (B) metformin, (C) olaparib (upper panel depicts wound healing assay after treatments and the graph below its quantification), (D and E) phenformin‐olaparib (F and G) and metformin‐olaparib (left panel depicts wound healing assay after treatments and the right graph its quantification). Data represent the mean ± SEM of triplicate of three independent experiments, statistical significance is represented by *(*P* < .05)

The antimigratory and anti‐invasion properties of phenformin, metformin and olaparib were examined using wound healing assays. A2780PAR and A2780CR cells were incubated for 24 hours with different doses of the phenformin (0‐1 mmol/L) (Figure 3A), metformin (0‐5 mmol/L) (Figure 3B) and olaparib (0‐2 µmol/L) (Figure 3C). After 24 hours, A2780PAR cells showed a high reduction in migration compared with the drug‐resistant cell line after treatment (~45% in single treatments, metformin 5 mmol/L *P* < .0073, phenformin 1 mmol/L *P* < .0022 and olaparib 2 µmol/L *P* < .0161). Furthermore, we examined the effects of the biguanides in combination with olaparib. Interestingly, we observed that the effects of both biguanides, phenformin (Figure 3D‐E) and metformin (Figure 3F‐G) are potentiated by the addition of olaparib to inhibit migration. Our results showed that A2780PAR cells showed a decrease of migration in a dose‐dependent manner (~80%metformin *P* < .0403, ~85% phenformin *P* < .0303). Moreover, A2780CR migration showed a similar decrement compared with parental cells after the cotreatment of biguanide‐olaparib (0.5 µmol/L) (~80%metformin *P* < .0029, ~81% phenformin *P* < .0156).

### 3.4 Combination of biguanides and olaparib enhances the expression of e‐cadherin and diminishes mesenchymal markers in oc cells

**Figure 4:**
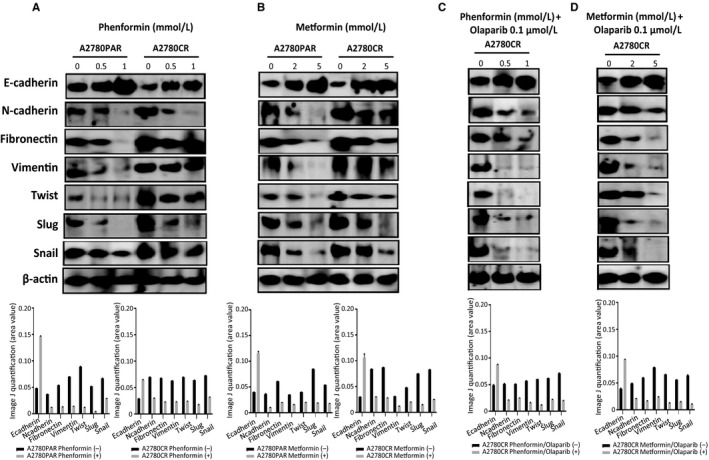
Biguanide treatment inhibits the expression of EMT markers. Protein expression levels of mesenchymal markers (Slug, Snail, Vimentin, N‐cadherin, Twist and Fibronectin) and the epithelial marker E‐cadherin in A2780PAR and A2780CR ovarian cancer cells treated for 72 hours with (A) phenformin, (B) metformin, (C) phenformin‐olaparib, (D) metformin‐olaparib. Bands were quantified using Image J software and normalized to β‐actin. Data represent the mean ± SEM of triplicate of three independent experiments

In order to investigate the ability of biguanides in regulating the expression of EMT markers including transcription factors (Twist, Snail and Slug) as well mesenchymal markers (N‐cadherin, fibronectin and vimentin). As shown in Figure 4A,B, we observed the down regulation of mesenchymal markers examined in A2780PAR and its resistant clone A2780CR cells following phenformin and metformin treatment. On the other hand, the epithelial marker E‐cadherin was significantly up regulated by biguanides, especially phenformin (*P* < .020). In addition, we examined the effects of the biguanides in combination with olaparib in A2780CR cells (Figure 4C,D). We found a dose‐dependent down regulation in EMT drivers (Twist‐1, snail‐1, and slug) (*P* < .016). Together, these results indicate that the combined treatment with biguanides/olaparib reverses the functional features of OC aggressive behavior by increased epithelial markers (E‐cadherin) with a concomitant reduction of mesenchymal markers (N‐cadherin, fibronectin and vimentin) at the protein level.

### 3.5 Snail knock‐down attenuates cell proliferation and migration in OC cells treated with biguanides

**Figure 5:**
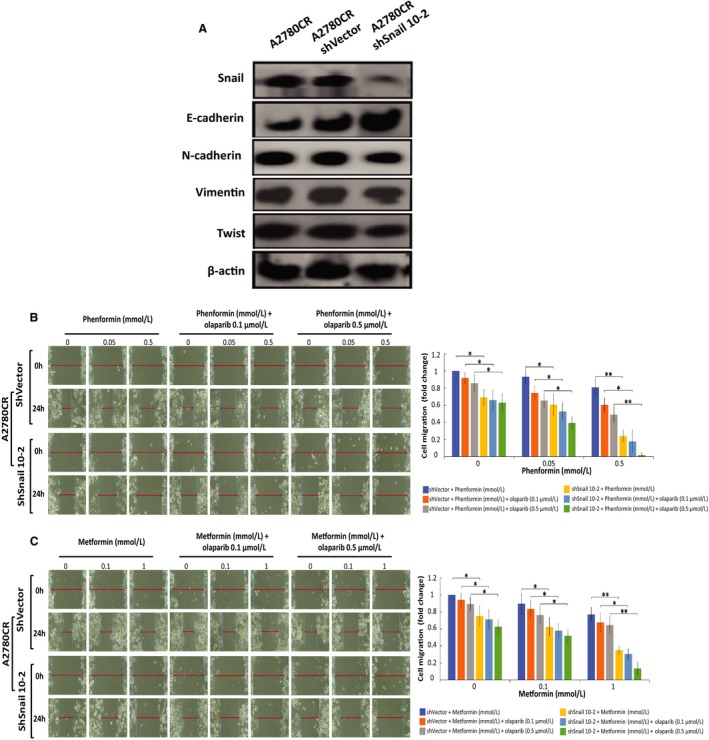
Cell migration is inhibited upon Snail knock‐down and enhanced by biguanide treatment. (A) Protein expression levels of mesenchymal markers (Snail, Vimentin, N‐cadherin and Twist) and E‐cadherin in A2780CR, A2780CR/shVector and A2780CR/shSnail 10‐2 ovarian cancer cells. A2780CR/shVector, A2780CR/shSnail 10‐2 ovarian cancer cells were treated for 24 hours and wound healing assay was performed to evaluate cell migration: (B) phenformin, phenformin‐olaparib 0.1 µmol/L and phenformin‐olaparib 0.5 µmol/L. (C) Metformin, metformin‐olaparib 0.1 µg, and metformin‐olaparib 0.5 µmol/L. Data represent the mean ± SEM for triplicate of three independent experiments, statistical significance is represented by *(*P* < 0.05) and **(*P* < 0.01).

The transcription factor Snail plays a crucial role in the modulation of initial steps of the epithelial‐mesenchymal process in which epithelial marker down regulation is critical. In this study, we showed that knock‐down of Snail was an additional strategy to suppress invasiveness and survival. As shown in Figure 5A, Snail was successfully down regulated in A2780CR‐shSnail 10‐2 cells as compared to controls. We found that on knock‐down of Snail, E‐cadherin was increased in A2780CR‐shSnail 10‐2 cells in comparison with vector control. Furthermore, cell migration was examined using the wound healing assay. We found that phenformin (Figure 5B) and metformin (Figure 5C) as single agents were able to decrease the migratory capacity of OC cells. Moreover, biguanide‐olaparib treatment showed a significant decrease in the migratory capacity of A2780CR‐shVector control (30%‐48%, *P* < .05). However this inhibition was exacerbated by the down regulation of Snail by ~90% in A2780CR‐shSnail 10‐2 cells treated with either metformin or phenformin in combination with olaparib 0.5 µmol/L as compared with shVector control (*P* = .0031, *P* = .0005 accordingly).

**Figure 6:**
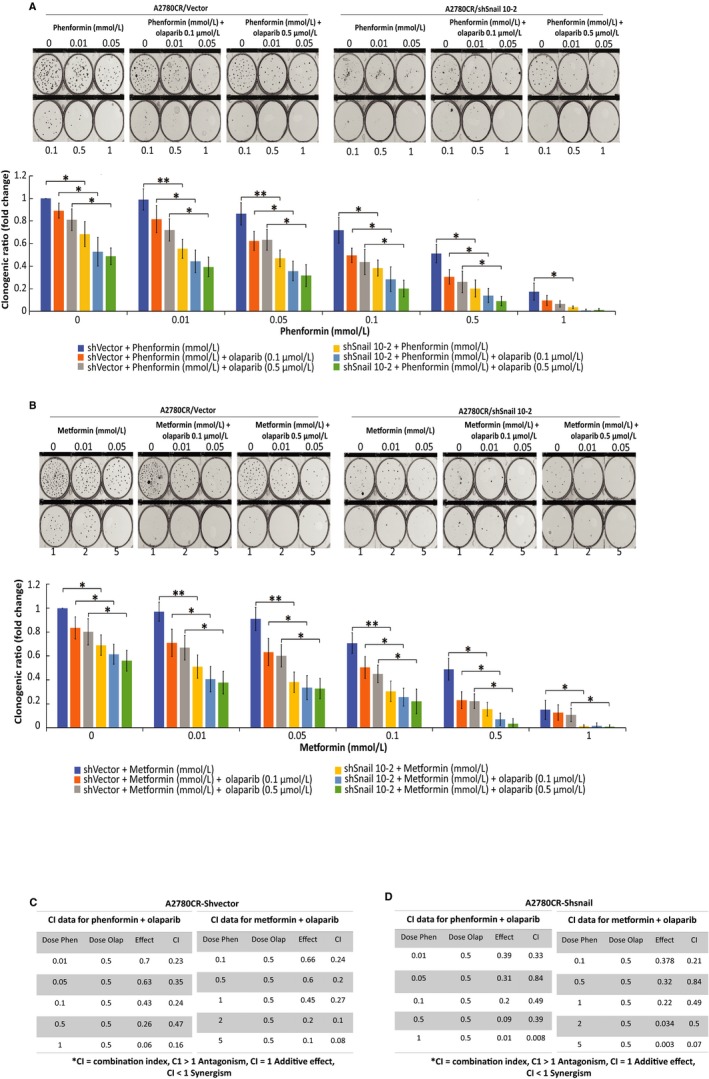
Cell survival is inhibited upon Snail knock‐down and enhanced by biguanide treatment. A2780CR/shVector, A2780CR/shSnail 10‐2 ovarian cancer cells were treated for 7 days with biguanides alone or in combination with olaparib and survival was determined using clonogenic assays. (A) Phenformin, phenformin‐olaparib 0.1 µmol/L and phenformin‐olaparib 0.5 µmol/L. (B) Metformin, metformin‐olaparib 0.1 µmol/L, and metformin‐olaparib 0.5 µmol/L. The evaluation of combination index for A2780CR/shVector (C) or A2780CR/shSnail 10‐2, (D) treated with phenformin or metformin and olaparib was calculated where CI < 1 indicates synergy between the two drugs and CI > 1 indicates an additive effect. Data represent the mean ± SEM for triplicate of three independent experiments, statistical significance is represented by *(*P* < .05) and **(*P* < .01)

Next, we evaluated cell proliferationon Snail knock‐down using colony formation assays. Phenformin (Figure 6A) or metformin (Figure 6B) induced a significant dose‐dependent inhibition of colony formation in A2780CR‐shSnail 10‐2 cells as compared to A2780CR‐shVector (*P* < .0182, *P* < .0202 accordingly). This colony formation was significantly decreased by the biguanide‐olaparib combination. Furthermore, we determined the nature of the interaction between both drugs, showing a high synergistic effect (CI < 1) (Figure 6C,D).

## 4 DISCUSSION

Ovarian cancer exhibits a high rate of platinum sensitivity in the first‐line setting, but resistance frequently develops in recurrent diseases.^23^ For that reason, understanding the underlying mechanism is critical for the development of new treatment options.

Epithelial‐mesenchymal transition (EMT) is regarded as a crucial contributing factor to cancer progression. Diverse factors have been identified as potent EMT inducers in ovarian cancer. Signals triggered by transcription factors such as Snail, Slug and Twist, diminish the expression of epithelial‐related genes such as E‐cadherin and at the same time, and enhance the expression of mesenchymal‐related genes such as vimentin.^24^ Like other epithelial‐derived tumors, extensive evidences have demonstrated EMT as a critical step for ovarian cancer progression.^25^ Immunohistological analyses of both primary and metastatic ovarian carcinoma reveal that EMT is significantly associated with peritoneal metastasis and survival of ovarian cancer patients.^26^ The correlation between EMT and aggressiveness of ovarian cancer is also supported by gene expression‐based studies in which metastatic tumors generally exhibit mesenchymal signatures.^27^

Poly (ADP ribose) polymerase (PARP) inhibitors (PARPi) are approved targeted therapeutics for breast and ovarian cancers bearing a germline *BRCA1/2* mutation. PARP‐1/‐2 are key enzymes in the DNA repair pathway which are targeted by PARPi. This inhibition confers efficacy to the treatment and induce cancer cell sensitization to conventional therapies.^28^ Moreover, PARP‐1 was shown to be prometastatic in melanoma in vitro and in vivo through vimentin‐induced E‐cadherin down regulation, Snail activation and consequently increased cell motility and migration.^29^ In addition, PARP‐1 silencing in prostate cancer promoted E‐cadherin expression and suppressed GSK‐3β phosphorylation, a crucial step in EMT signaling.^30^ This indicates that PARP silencing or maybe even PARP inhibition may have the efficacy of blocking EMT and attenuating metastasis.

Biguanides are anti‐diabetic medications that have gained significant attention as anticancer drugs. Moreover, metformin has been proved to suppress transforming growth factor β pathway and reverse EMT^31^ or inhibit multidrug resistance protein 1 expression and reverse chemoresistance in hepatocellular carcinoma.^32^ Previously, we have reported a pilot trial consistent with the anti‐proliferative effects of metformin which showed an increasem in E‐cadherin expression levels while suppressinge the EMT transition in women with endometrial cancer.^33^, ^34^ Furthermore, we have published that metformin induced apoptosis in ovarian cancer cells in an AMPK‐independent manner and provoked a cell cycle arrest in the S and G2/M phase. In addition, we established that metformin coulda induce apoptosis by activating caspases 3/7, down‐regulating Bcl‐2 and Bcl‐xL expression, and up‐regulating Bax and Bad expression. The induction of apoptosis by metformin was also enhanced by cisplatin.^17^ Altogether these studies suggest the anti‐tumor efficacy of metformin on cancer cell proliferation,^35^ EMT and drug‐resistance. Phenformin, as a derivative of metformin, shows more potent efficacy than metformin in inhibiting tumor growth. A recent study has shown phenformin to inhibit ovarian cancer tumor growth in vitro and in vivo as well as induction of cell cycle arrest and apoptosis (Jackson et al, 2017).

In thise present study, we aimed to investigate the possible use of biguanides in combination with PARPi. Our results indicated that the migration and clonogenicity of both A2780 parental and its resistance daughter clone cells were evidently suppressed by metformin, phenformin and olaparib as single treatments in a dose‐dependent manner. Furthermore, combination of these drugs further potentiates the effect in these cells. We have used the olaparib in the minimal dose levels that synergistically work with biguanides to suppress the cell survival and migration in A2780 cells. These results supports that this combinatorial approach was effective with lower doses. Therefore, a question was posed, what is the mechanism involved in the sensitization induced by low dose of olaparib?

EMT is a promoter of invasion and metastasis in many tumors^36^, ^37^ and the inducer of malignant transformation.^38^ The Snail family includes EMT key regulators playing important roles in multiple pathways that trigger mesenchymal transformation. Several studies have shown tumor growth, proliferation and metastasis to be attenuated by Snail knock‐down.^39^, ^40^ Therefore, Snail is considered to be a therapeutic target for the treatment of advanced and drug‐resistant cancers.

In our study, in order to find key targets in olaparib‐induced sensitization of epithelial ovarian cancer A2780CR cells that were resistant to biguanide treatment, Snail knock‐down was performed. The efficacy of Snail knock‐down cells treated with biguanides and olaparib was examined. We observed lower cell migration and clonogenicity capacity in A2780CR‐shSnail cells compared with A2780CR‐shVector cells. These results let us propose that Snail is a key target in olaparib‐induced sensitization that deserves more attention as part of the combination therapy.

## 5 CONCLUSIONS

Nowadays, the interest in combining targeted inhibitors with common metabolic inhibitors to hinder resistance is emerging. Biguanides, metformin and phenformin, are widely used for treatment of patients with type 2 diabetes, however, there is evidence of its anticancer effects in the literature. Recent studies highlight the direct cellular benefits of combining biguanides with current targeted therapy. Here, our study demonstrates that biguanides in combination with PARPi synergistically reduce EMT, proliferation and survival of ovarian drug‐resistant cancer cells. Moreover, we provide further evidence of such synergisms that can potentially be useful in ovarian cancer treatment and is a rationale and cost‐effective strategy to improve outcomes in cancer patients treated with targeted therapies. Further investigation into the effectiveness of this combination in vivo may lead to widespread treatment opportunities in the future for ovarian cancer patients.

## CONFLICT OF INTEREST

The authors have no potential conflict of interest.

## AUTHOR CONTRIBUTION

AY designed the research, supervised the study and helped in drafting the manuscript. QW and VLO contributed equally to this work. QW, JB, TB, VLO, OA, IL were involved in the acquisition of the data, analysis of the results and performed the experiments. VLO wrote the manuscript. All authors made substantial contributions to the conception and design, acquisition of the data, analysis and interpretation of the data, all authors were involved in revising and critically evaluating the manuscript for important intellectual content. In addition, each author participated sufficiently in the work to take public responsibility for appropriate portions of the content and agreed to be accountable for all aspects of the work in ensuring that questions related to the accuracy or integrity of any part of the work are appropriately investigated and resolved.
